# Multimodality imaging findings of hematological malignancies involving the breast: a retrospective analysis based on the updated BI-RADS v2025 Manual

**DOI:** 10.1007/s11845-026-04399-8

**Published:** 2026-04-22

**Authors:** Umur Anil Pehlivan, Hulya Ozdemir

**Affiliations:** Department of Radiology, Başkent University Faculty of Medicine, Adana Dr. Turgut Noyan Application and Research Center, Adana, Türkiye

**Keywords:** Breast neoplasms, Elasticity imaging techniques, Lymphoma, Magnetic resonance imaging, Ultrasonography

## Abstract

**Background:**

Hematological malignancies involving the breast are rare and can mimic primary breast carcinomas. The recent release of the ACR BI-RADS v2025 Manual introduces updated descriptors that may enhance the characterization of breast lesions.

**Aims:**

This study aims to characterize the multimodality imaging features of histopathologically proven breast involvement in lymphoma, leukemia, and plasmacytoma, specifically utilizing the BI-RADS v2025 Manual.

**Methods:**

We retrospectively analyzed the clinical and radiological data of 25 patients with primary or secondary hematological breast involvement. Imaging findings from mammography (MG), ultrasound (US), elastography, and magnetic resonance imaging (MRI) were evaluated.

**Results:**

The cohort (mean age: 50.4 ± 17.6 years) showed a high prevalence of secondary involvement (96%), multifocal (64%), and bilateral (36%) disease. On MG, an equal-to-high-density mass with a lobulated shape and obscured margins, without associated microcalcifications, was the predominant finding. US most frequently demonstrated a lobulated mass with indistinct margin, heterogeneous echotexture, and parallel orientation, often accompanied by an echogenic rind and posterior acoustic enhancement. On elastography, approximately 60% of lesions exhibited intermediate stiffness. MRI typically revealed T2-hyperintense mass with heterogeneous contrast enhancement and marked diffusion restriction (low ADC values). Axillary lymphadenopathy was present in 52% of the cases.

**Conclusion:**

Hematologic malignancies typically present as lobulated shape, non-calcified masses with obscured or indistinct margins, intermediate elasticity, and marked diffusion restriction. Awareness of these patterns within the BI-RADS v2025 framework is essential for achieving a precise diagnosis and will prevent unnecessary surgical interventions.

## Introduction

Hematological malignancies account for 6.6% of all new cancer diagnoses and 7.2% of cancer-related deaths worldwide [1]. Despite the significant incidence of this disease spectrum—which encompasses lymphomas, plasmacytomas/multiple myeloma, and leukemias—breast involvement remains exceedingly rare, representing less than 1% of all breast tumors [2, 3]. The most common pathological entity within this spectrum is lymphoma, of which diffuse large B-cell lymphoma, a non-Hodgkin lymphoma subtype, is the most prevalent histological subgroup [2]. Non-lymphomatous pathologies, such as leukemias and plasmacytomas, are chiefly characterized in the literature through case reports and small series [4, 5]. In these rare scenarios, a prior hematological diagnosis can streamline the identification of secondary breast lesions; however, breast manifestations may also emerge as the sentinel sign of an underlying occult systemic process [2, 6]. While secondary involvement is the more frequent clinical presentation, these pathologies can manifest as isolated involvement of the breast and/or regional lymph nodes, consistent with the clinical presentation of primary disease [2, 7]. While the typical presentation consists of a solitary unilateral lesion, bilateral and multifocal involvement may also be encountered [5, 7–10].

Beyond the clinical challenge, the diagnostic process is further complicated by the inherently non-specific radiological features of these diseases, which exhibit a wide spectrum of imaging findings that often mimic both benign and malignant entities [2, 7, 8, 11]. Breast involvement of hematological disease represents a fundamentally systemic process and is therefore managed primarily with systemic therapy, with or without radiotherapy, rather than surgical excision [3, 12]. Consequently, histopathological evaluation remains an indispensable component in establishing a definitive diagnosis [13]. Accordingly, clinical awareness and familiarity with the radiological characteristics of these rare entities are vital to prevent unnecessary surgical interventions.

While the current literature includes several studies regarding breast involvement in hematological malignancies, some variabilities exist in reported outcomes and descriptor interpretations [8–11, 14]. These variabilities hinder the ability to generalize the specific imaging characteristics of the disease. Following the recent release of the American College of Radiology (ACR) Breast Imaging Reporting and Data System (BI-RADS) v2025 Manual, minor yet significant updates have been implemented in the guidelines. In the ACR BI-RADS v2025 mammography (MG) section, the shape descriptor lobulated has been reintroduced to provide greater clarity, while the margin term microlobulated has been removed and replaced by the descriptor indistinct to minimize diagnostic confusion [15]. In the ultrasound (US) section, the updated BI-RADS v2025 Manual has introduced a new lobulated shape sub-descriptor. Additionally, new descriptors have been added to characterize the perilesional tissue, specifically incorporating echogenic pseudocapsule and echogenic rind as associated sonographic features [15]. Furthermore, according to the BI-RADS v2025 magnetic resonance imaging (MRI) section, T2-signal intensity has been formally added as a new sub-descriptor for mass lesions, reflecting its increasing clinical importance in MRI evaluations [15]. To our knowledge, no study has yet evaluated hematological breast involvement specifically through the lens of the updated BI-RADS v2025 descriptors. Owing to this gap in the literature, the aim of this study is to present our institutional experience and multimodality imaging findings of hematological malignancies’ breast involvement, characterized according to the ACR BI-RADS v2025 Manual.

## Materials and methods

### Study design and ethical approval

This descriptive, retrospective single-center study was conducted at the Department of Radiology, Division of Breast Imaging, Başkent University Faculty of Medicine Adana Dr. Turgut Noyan Application and Research Center. The study protocol was approved by the institutional non-interventional clinical research ethics committee (Project No: KA25/494, approval date: 06.01.2026). The study was carried out in accordance with the principles of the Declaration of Helsinki.

## Patient population

**Fig. 1:**
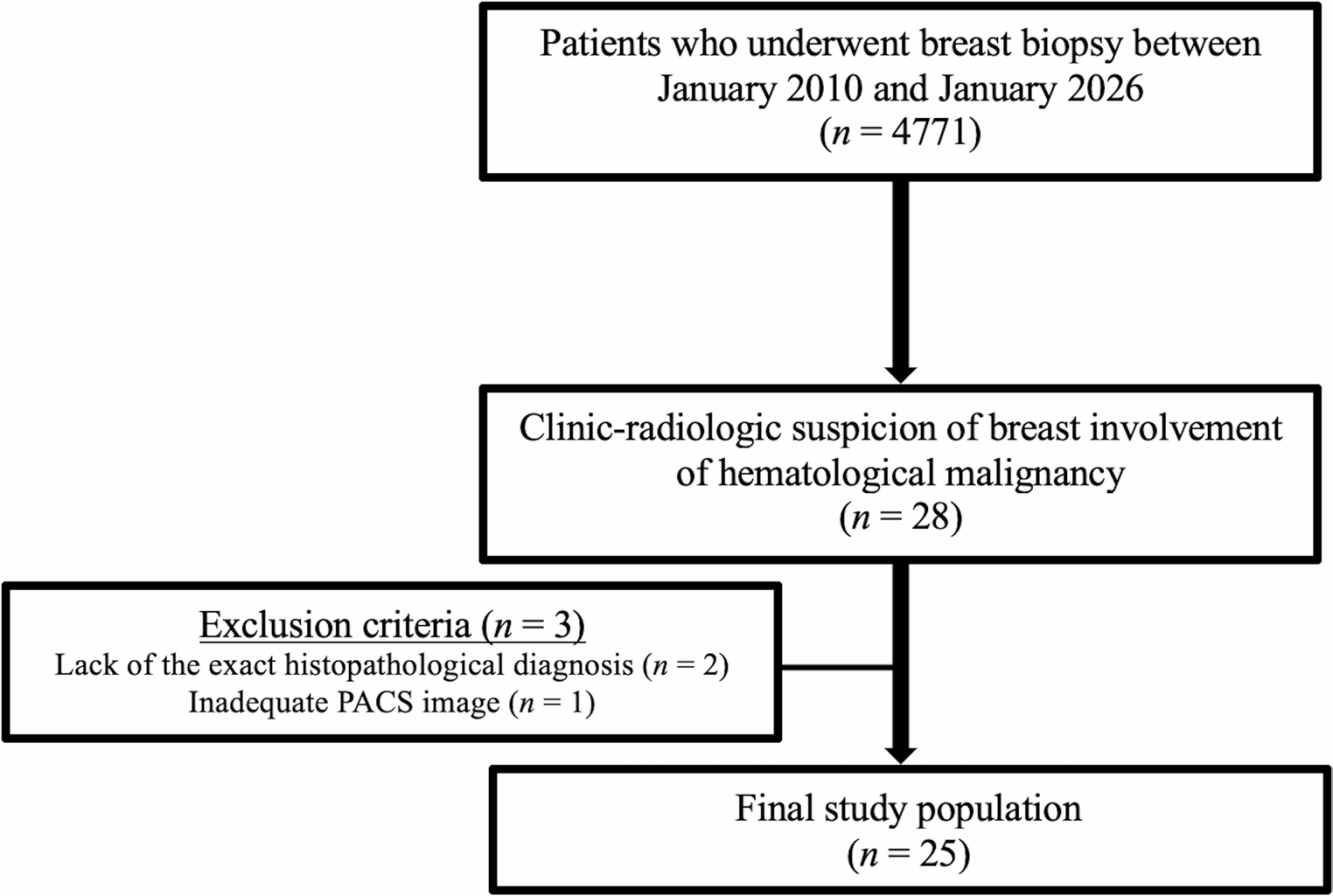
The study flowchart. PACS, Picture Archiving and Communication System

A total of 4,771 patients underwent US-guided breast core needle biopsy procedures at our breast center between January 2010 and January 2026. The inclusion criteria were clinic-radiologic suspicion of hematological malignancy involving the breast. Three patients were excluded: two lacked definitive histopathological verification, and one was excluded due to inadequate retrospective Picture Archiving and Communication System (PACS) images for comprehensive re-evaluation. Consequently, the final study population consisted of 25 patients with confirmed histopathological diagnoses (Fig. 1).

## Demographic and clinical data

Demographic characteristics, including age and sex, along with clinical data such as primary versus secondary disease involvement and histopathological subtypes, were retrieved from the hospital’s electronic information system.

## Imaging protocols and technical equipment

All imaging data were retrospectively retrieved and analyzed via the PACS. MG evaluations in craniocaudal and mediolateral oblique projections were available for 13 patients; these were performed using a Senographe Pristina (GE Healthcare, Buc, France) or a Mammomat Inspiration (Siemens Healthineers, Erlangen, Germany). US was performed on all patients using two identical Acuson S2000 systems (Siemens Healthineers, Erlangen, Germany), utilizing shear-wave and/or strain elastography. Color and/or power Doppler US was also performed in 15 patients. MRI, including dynamic contrast-enhanced and diffusion-weighted sequences, was conducted in three patients using either a 1.5 Tesla MAGNETOM Avanto or a 3 Tesla MAGNETOM Skyra system (Siemens Healthineers, Erlangen, Germany).

## Image analysis and evaluation

All radiological images were retrospectively re-evaluated by two radiologists holding the European Diploma in Radiology (EDiR) certification, with all findings reached through consensus based on ACR BI-RADS v2025 Manual. The assessment included lesion lateralization, lesion count (solitary, < 5, or multiple), the presence of axillary lymph node involvement (negative or positive), and signs of cutaneous involvement. The biopsied lesion was assigned as the dominant lesion, and its maximum diameter was recorded. The apparent diffusion coefficient (ADC) value and time-intensity curve assessment were performed via standard software (SyngoVia, Siemens Healthineers, Erlangen, Germany). Final lesion classification and BI-RADS category assignment were performed based on the imaging features of the dominant lesion according to the ACR BI-RADS v2025 Manual [15].

### Statistical analysis

Continuous variables were expressed as mean ± standard deviation for normally distributed data, while non-normally distributed variables were presented as median (min-max). Categorical variables were analyzed using frequencies and percentages. A p-value of less than 0.05 was considered statistically significant.

## Results

The study population consisted of 25 patients with various hematological malignancies, including mature B-cell lymphoma (*n* = 17), T-cell lymphoblastic leukemia (*n* = 3), and plasmacytoma (*n* = 2), alongside individual cases of extranodal marginal zone lymphoma, peripheral T-cell lymphoma, and precursor B-cell lymphoblastic lymphoma. While one case presented with primary B-cell lymphoma, the remaining cases represented as secondary breast involvement. The overall mean age was 50.4 ± 17.6 years (range: 20–83). Specifically, the mean age in the lymphoma group was 53.7 ± 16.7 years, while the ages of the leukemia patients were 20, 20, and 49 years, and the plasmacytoma patients were 42 and 55 years old.

Unilateral involvement was observed in 16 patients, while nine exhibited bilateral distribution. Within the lymphoma subgroup (*n* = 20), 13 cases were unilateral and seven were bilateral; notably, all bilateral cases presented with multiple lesions. Axillary lymphadenopathy was identified in 12 patients with lymphoma and one with leukemia, whereas it was absent in both plasmacytoma cases. Five mature B-cell lymphoma patients presented with concomitant skin thickening. Clinical and demographic characteristics are summarized in Table 1.

**Table 1:** Clinical characteristics and disease distribution of patients with breast involvement of hematological malignancies

Case	Age	Diagnosis	Pattern (Primary/Secondary)	Size (mm)	Side	Number of Lesions	Axillary LAP	Skin Thickening
1	58	B-cell lymphoma	Secondary	8	Right	3 lesions	Negative	Negative
2	72	B-cell lymphoma	Secondary	17	Bilateral	Multiple	Positive	Negative
3	26	B-cell lymphoma	Secondary	60	Bilateral	Multiple	Positive	Positive
4	36	B-cell lymphoma	Secondary	9	Bilateral	Multiple	Negative	Negative
5	73	B-cell lymphoma	Secondary	50	Bilateral	Multiple	Positive	Positive
6	72	B-cell lymphoma	Secondary	16	Bilateral	Multiple	Positive	Negative
7	28	B-cell lymphoma	Secondary	36	Bilateral	Multiple	Negative	Positive
8	33	B-cell lymphoma	Secondary	110	Left	Multiple	Positive	Positive
9	69	B-cell lymphoma	Secondary	25	Bilateral	Multiple	Positive	Negative
10	40	B-cell lymphoma	Secondary	100	Right	Multiple	Positive	Positive
11	61	B-cell lymphoma	Secondary	21	Left	Solitary	Negative	Negative
12	41	B-cell lymphoma	Secondary	39	Right	Solitary	Positive	Negative
13	69	B-cell lymphoma	Secondary	18	Left	Solitary	Negative	Negative
14	42	B-cell lymphoma	Primary	50	Left	Solitary	Positive	Negative
15	63	B-cell lymphoma	Secondary	18	Left	Solitary	Negative	Negative
16	83	B-cell lymphoma	Secondary	56	Left	Solitary	Positive	Negative
17	46	B-cell lymphoma	Secondary	48	Left	Solitary	Negative	Negative
18	58	Extranodal marginal zone lymphoma	Secondary	45	Right	3 lesions	Positive	Negative
19	57	Peripheral T-cell lymphoma	Secondary	10	Right	2 lesions	Positive	Negative
20	47	Precursor B-cell lymphoma	Secondary	24	Right	Multiple	Negative	Negative
21	55	Plasmacytoma	Secondary	50	Right	2 lesions	Negative	Negative
22	42	Plasmacytoma	Secondary	38	Left	Solitary	Negative	Negative
23	20	T-cell lymphoblastic leukemia	Secondary	25	Bilateral	Multiple	Negative	Negative
24	20	T-cell lymphoblastic leukemia	Secondary	80	Bilateral	Multiple	Positive	Negative
25	49	T-cell lymphoblastic leukemia	Secondary	110	Left	Solitary	Negative	Negative

**Fig. 2:**
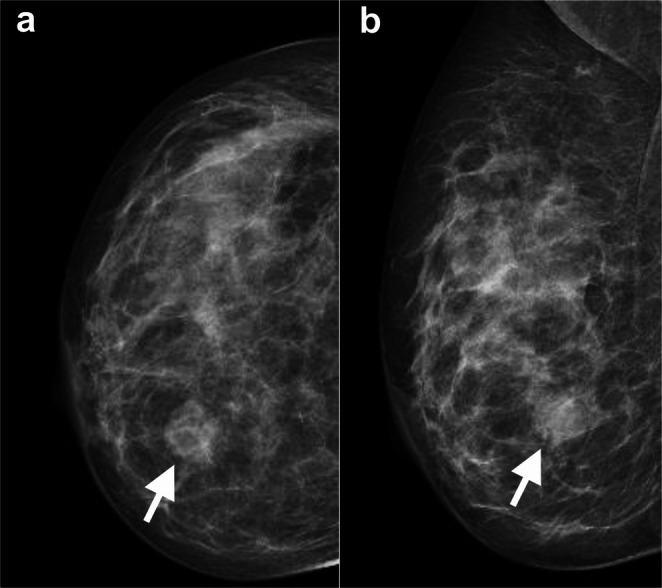
Precursor B-cell lymphoma in a 47-year-old woman. (**a**) Craniocaudal and (**b**) mediolateral oblique mammograms demonstrate a lobulated, equal-density mass with obscured margins and no associated microcalcifications in the inner-central quadrant of the right breast (arrows)

The median size of the dominant lesions was 38 mm (range: 8–110 mm). On MG, the most frequent finding was mass lesion (*n* = 11) (Fig. 2). The most common mass shapes were lobulated (*n* = 5), followed by oval (*n* = 3), irregular (*n* = 2) and round (*n* = 1). Mass margins were predominantly obscured (*n* = 7), followed by indistinct (*n* = 2) and circumscribed (*n* = 2). High-density masses were identified in six cases, whereas equal density was noted in five. Additionally, focal asymmetry was observed in the remaining two cases. Notably, calcifications were absent in 12 patients. Only one patient, diagnosed with extranodal marginal zone lymphoma, had a solitary coarse calcification.

**Fig. 3:**
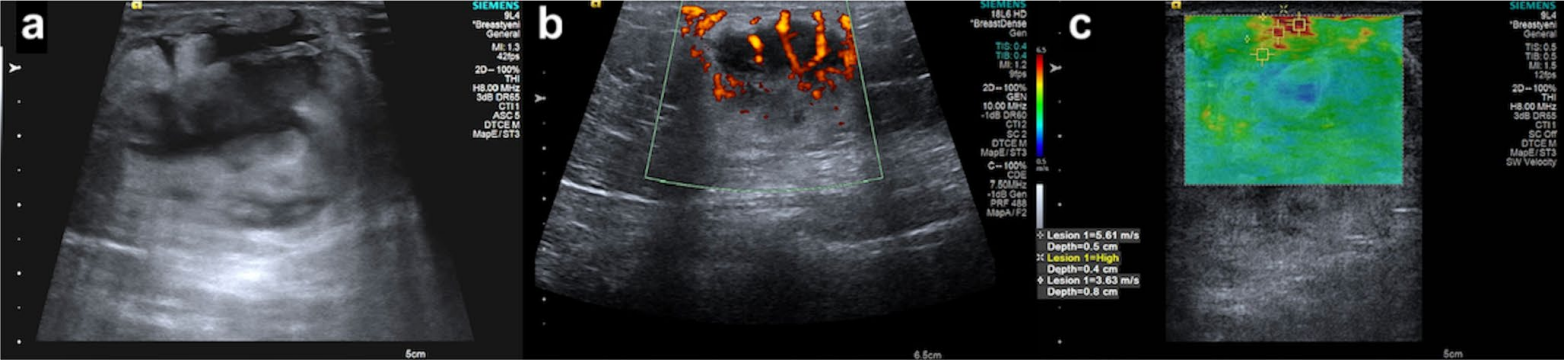
Breast plasmacytoma in a 55-year-old woman. (**a**) B-mode ultrasound image demonstrates a lobulated-shape mass with indistinct margins, heterogeneous echotexture, parallel orientation, and a prominent echogenic rind. Marked posterior acoustic enhancement is also observed. (**b**) Power Doppler ultrasound image reveals vascularity within the lesion. (**c**) Shear-wave elastography image demonstrates high tissue stiffness

All dominant lesions were identified as masses on US (Fig. 3). The most frequent shape was lobulated (*n* = 12), followed by oval (*n* = 9) and round (*n* = 4). Lesion margins were most commonly indistinct (*n* = 17), followed by circumscribed (*n* = 3), angular (*n* = 3), and microlobulated (*n* = 2). The internal echo pattern was predominantly heterogeneous (*n* = 15), with hypoechoic (*n* = 9) and hyperechoic (*n* = 1) patterns being less common. Eight dominant lesions demonstrated no posterior acoustic features, whereas posterior acoustic enhancement was observed in 17 lesions. Regarding orientation, 19 masses (76%) exhibited a parallel orientation, while six (24%) were non-parallel. An echogenic pseudocapsule was present in one dominant lesion, while an echogenic rind was identified in 13 lesions. Doppler US revealed internal and/or peripheral vascularity in 14 of the 15 evaluable cases. Elastography findings were classified as intermediate in 15 lesions, hard in six lesions, and soft in four.

**Fig. 4:**
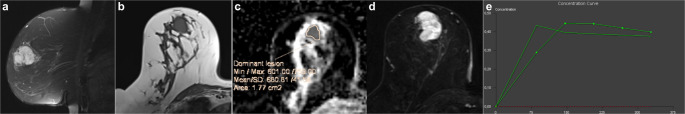
Extranodal marginal zone lymphoma in a 58-year-old woman. (**a**) Sagittal fat-suppressed T2-weighted and (**b**) axial precontrast T1-weighted images demonstrate a lobulated-shaped T2-hyperintense mass with indistinct margins in the right breast. (**c**) The apparent diffusion coefficient (ADC) map shows low ADC values, indicating diffusion restriction. (**d**) Axial postcontrast subtraction T1-weighted image demonstrates heterogeneous enhancement. (**e**) The time-intensity curve shows moderate early enhancement followed by a plateau pattern in the delayed phase

**Fig. 5:**
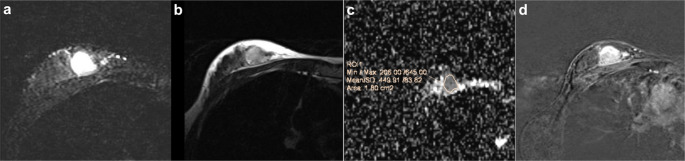
T-cell lymphoblastic leukemia in a 20-year-old woman. (**a**) Fat-suppressed and (**b**) non–fat-suppressed T2-weighted images demonstrate an oval-shaped T2-hyperintense mass with circumscribed margins in the right breast. (**c**) The apparent diffusion coefficient (ADC) map demonstrates marked diffusion restriction with very low ADC values, reflecting high tumor cellularity. (**d**) Axial postcontrast subtraction T1-weighted image reveals heterogeneous enhancement pattern

All dominant lesions were classified as mass lesions on MRI, demonstrating T1 hypointensity and T2 hyperintensity. Morphologically, both the extranodal marginal zone lymphoma and mature B-cell lymphoma lesions exhibited lobulated shapes with indistinct margins (Fig. 4). In contrast, the dominant lesion in the patient with T-cell lymphoblastic leukemia appeared as an oval mass with circumscribed margins (Fig. 5). Dynamic contrast-enhanced MRI demonstrated heterogenous contrast enhancement pattern with two distinct kinetic patterns. The lesions diagnosed with extranodal marginal zone lymphoma and mature B-cell lymphoma showed moderate early enhancement followed by a plateau in the delayed phase. The lesion associated with T-cell lymphoblastic leukemia showed moderate early enhancement with persistent delayed enhancement. All lesions demonstrated marked diffusion restriction, with very low ADC values of 0.680, 0.450, and 0.430 × 10⁻³ mm²/s, respectively.

Regarding the BI-RADS assessment, the most frequent classification was Category 4B (*n* = 12), followed by 4C (*n* = 8), 4A (*n* = 3), 3 (*n* = 1), and 5 (*n* = 1).

## Discussion

This study is the first to evaluate histopathologically proven breast involvement in hematological malignancies using the recently updated ACR BI-RADS v2025 Manual. In our cohort, the predominant mammographic presentation was a high-density, lobulated mass with obscured margins, characterized by a notable absence of calcifications. Sonographically, these lesions typically appeared as lobulated masses with indistinct margins and a parallel orientation, frequently accompanied by an echogenic rind, posterior acoustic enhancement, and internal and/or peripheral vascularity. MRI further characterized these entities as T2-hyperintense masses showing heterogeneous contrast enhancement and markedly restricted diffusion. Our findings suggest that this specific constellation of imaging features, particularly the lobulated shape with obscured-indistinct margin, the presence of an echogenic rind, the absence of microcalcifications, intermediate elasticity, and markedly low ADC values serve as crucial diagnostic clues.

The mean age in the current cohort (50.4 years) was lower than in some multicenter series [10, 14], but remained consistent with the middle-age predominance reported in the broader literature [4, 5, 8, 9, 11, 16]. Notably, leukemia patients were diagnosed at a younger age than those with lymphoma or plasmacytoma, a finding corroborated by existing leukemia-specific series [5, 11]. This age discrepancy likely reflects the inherent biological differences and distinct peak incidence periods of acute leukemias compared to more chronic lymphoid malignancies.

Secondary involvement was observed in nearly all patients (24/25). Consistent with the general consensus that breast lymphomas are typically secondary manifestations [2, 9], 19 of our 20 lymphoma cases were secondary. Although some series have reported on unexpectedly high rate of primary breast lymphoma (61–86.6%) [10, 16, 17], our findings align with broader clinical trends. Similarly, both plasmacytoma cases occurred during multiple myeloma follow-up and this 100% secondary rate mirrors large-scale studies [4]. Regarding leukemia, despite reported primary rates of 17.3%–25% [9, 11], all our cases were secondary. This finding may be attributed to our institution’s role as a tertiary reference center which predominantly evaluates advanced or relapsed leukemia cases in which systemic dissemination is more frequent [2, 18].

In our study, bilateral involvement was observed in 36% of the overall cohort and 35% of the lymphoma subgroup; these findings are consistent with the 20–28% rates reported in the literature [9, 10, 14]. Crucially, high bilaterality serves as a key diagnostic indicator for hematological malignancies, as this is notably rare in primary breast carcinomas [19]. Solitary lesions were identified in only 36% of our patients, which is lower than the 45–53% reported previously [9, 14]. This finding is likely due to the high rate of secondary involvement, generally presenting as multifocal disease [2, 10, 20].

Axillary lymphadenopathy was identified in 52% of our cohort. The high prevalence of prominent nodal disease in our series suggests that hematological malignancies present with more extensive lymphadenopathy than primary breast carcinomas, compatible with the literature [2, 8, 9]. Notably, skin thickening was observed exclusively in patients with mature B-cell lymphoma phenotypes. While the overall incidence of skin thickening in the entire lymphoma cohort (*n* = 20) was 25%, the prevalence was higher within the mature B-cell lymphoma subpopulation (5/17; 29.4%), though still lower than reported by Durhan et al. [9]. As noted by Glazebrook et al., features such as skin thickening in breast lymphomas are typically secondary to lymphatic obstruction rather than direct dermal invasion [6]. This discrepancy may reflect differences in the biological characteristics and subtype distribution among the studied cohorts.

Regarding MG characteristics, 84.6% of our patients presented with a mass, corroborating reported rates of 64%–80% [9, 14]. The reintroduction of the lobulated shape descriptor in the ACR BI-RADS v2025 MG section is a crucial update for these entities. In our study, the 45.5% lobulated rate reflects the expansive growth pattern of hematological tumors along low-resistance tissue planes. We hypothesize that the high frequency of irregular shapes reported in previous MG series likely represents what is now precisely defined as lobulated in the new manual. Historically, when lobulated was not available as a standalone descriptor, radiologists often defaulted to irregular to describe these complex lesions [5, 9, 14, 15]. This shift in the v2025 Manual allows for a clearer distinction between the circumscribed-but-multicentric growth of hematological malignancies and the truly irregular, invasive margins typical of epithelial carcinomas. Similarly, the high prevalence of obscured margins reflects the non-desmoplastic growth pattern; unlike the desmoplastic and infiltrative nature of primary carcinomas. These entities often respect anatomical structures, leading to margins concealed by adjacent fibroglandular tissue. One of the notable findings in our cohort was the mass density; six dominant lesions exhibited high density, while five showed equal density. Unlike many primary breast carcinomas that are typically high-density, the presence of equal-density (isodense) masses in a significant portion of our cases underscores a potential diagnostic pitfall. These lesions may blend with the surrounding fibroglandular tissue, potentially leading to false-negative evaluations, particularly in dense parenchymal backgrounds.

In our US outcomes, all lesions presented as masses, with lobulated (48%) and oval (36%) shapes predominating. The frequent classification of such masses as irregular in older series [5, 9, 14] likely stemmed from the absence of the lobulated descriptor in previous US lexicons. Utilizing the BI-RADS v2025 Manual, our 48% lobulated rate more accurately reflects the non-desmoplastic nature of these malignancies. Furthermore, the presence of indistinct margins in 17 cases likely represents a transitional zone where hematological cells subtly blend into the surrounding parenchyma without the overt desmoplastic reaction typical of epithelial carcinomas. Posterior acoustic enhancement was observed in 68% of lesions, aligning with the literature [9], while the absence of shadowing underscores the high cellular homogeneity. The high prevalence of the echogenic rind in our study, a feature generally associated with malignancy, underscores its additional diagnostic utility [15]. In hematological involvement, this finding can represent peritumoral edema or outcomes of the relation between rapid infiltrative growth and surrounding parenchyma. Internal and/or peripheral vascularity was detected in 93% of cases, corroborating the hypervascularity emphasized in major series [5, 10, 11, 14]. In our cohort, the predominance of intermediate stiffness (60%) on elastography serves as a vital diagnostic clue; unlike hard primary breast carcinomas, hematological malignancies tend to retain greater tissue elasticity due to their non-desmoplastic cellular infiltration [21, 22].

In our cohort, all patients who underwent MRI presented with mass lesions characterized by T1-hypointensity and T2-hyperintensity. These findings align with reports indicating that 91–95% of such cases present as T2-hyperintense masses [5, 14, 16]. Furthermore, MRI proved indispensable for assessing disease extent in patients with dense parenchyma (BI-RADS category C-D), where mammography showed limited sensitivity [11]. The most consistent MRI finding was profound diffusion restriction with markedly low ADC values. This parameter carries high diagnostic specificity, as ADC values in hematological malignancies, ranging between 0.43 and 0.68 × 10⁻³ mm²/s in this study, are significantly lower than the typical ranges reported for primary epithelial breast cancers. For instance, in a study utilizing multiple b-values, the mean ADC values for primary malignant breast lesions ranged from 0.83 ± 0.26 to 0.94 ± 0.25 × 10⁻³ mm²/s [23]. This profound diffusion restriction in hematological entities reflects their extremely high nuclear-to-cytoplasmic ratio and dense cellular packing, which typically exceeds that of invasive breast carcinomas. The two kinetic patterns observed in our cohort highlight the variable vascular and stromal characteristics of hematological malignancies involving the breast. Therefore, dynamic contrast enhanced MRI kinetics alone should not be considered a reliable discriminator in these cases, and interpretation must be integrated with other imaging features such as marked diffusion restriction, T2 hyperintensity, and the absence of spiculated margins.

Regarding the BI-RADS distribution of the present study, the majority of lesions were classified as 4B, followed by 4C, reflecting the intermediate-to-high suspicion profile of hematological breast malignancies. However, the presence of a BI-RADS 3 case and the observed circumscribed or lobulated morphology underscores a critical diagnostic pitfall inherent to this disease spectrum. In this BI-RADS 3 lesion, the predominantly hyperechoic heterogeneous echotexture, combined with a round shape and circumscribed margins, constituted a low-suspicion sonographic profile under both the ACR BI-RADS 5th Edition and the updated v2025 Manual. Although internal echo texture appeared heterogeneous, the absence of the vascularity on Doppler interrogation and the lack of additional high-suspicion features precluded upstaging of the BI-RADS category. Importantly, neither the 5th Edition nor the v2025 Manual mandates upstaging solely on the basis of heterogeneous echotexture in the absence of corroborating suspicious findings; thus, the lesion was consistently assessed as BI-RADS 3 across both lexicons. Unlike the spiculated margins and posterior acoustic shadowing characteristic of primary invasive breast carcinoma, hematological malignancies frequently exhibit pseudo-benign sonographic features, including lobulated shape, parallel orientation, circumscribed margins, and posterior acoustic enhancement, which collectively increases the risk of under-classification. Such an underestimation carries significant clinical consequences. An inappropriate recommendation for short-term follow-up instead of immediate biopsy can lead to a substantial delay in the initiation of essential systemic therapy. Therefore, we emphasize that multimodality ancillary findings, specifically the presence of an echogenic rind on US, intermediate tissue stiffness on elastography, markedly low ADC values on diffusion-weighted MRI, or a known underlying hematological diagnosis, should collectively prompt a high index of suspicion and upstaging to at least BI-RADS 4, even in morphologically circumscribed masses, to ensure timely and accurate diagnosis.

Despite its retrospective design and small sample size—inherent to the rarity of these malignancies—this study is the first to implement the BI-RADS v2025 Manual. However, as the present study is a descriptive in nature, these findings warrant validation in larger, prospective series. Although Contrast-Enhanced Mammography (CEM) is now a primary section in the BI-RADS v2025 Manual, our cohort lacked CEM data, representing a limitation for multi-modality correlation. Nevertheless, the inclusion of CEM in the new guidelines opens significant research avenues, and future studies should specifically investigate the CEM findings of hematological breast involvement. Furthermore, the prognostic value of specific markers, such as the echogenic rind or exceptionally low ADC values, remains to be correlated with systemic treatment response and overall survival. Finally, leveraging radiomics and deep learning, as demonstrated in recent US decision-support models, could provide objective quantification of the morphological features identified [24].

In conclusion, the current study demonstrates that hematological malignancies of the breast exhibit a specific constellation of imaging features—lobulated shape, obscured/indistinct margin, non-calcified masses, echogenic rinds, intermediate tissue stiffness, and markedly low ADC values—which facilitate their differentiation from primary breast carcinoma. Standardized reporting with the BI-RADS v2025 Manual may reduce diagnostic delays and prevent unnecessary surgical referrals in this clinically vulnerable population.
